# The role of acetyltransferases for the temporal-specific accessibility of β-catenin to the myogenic gene locus

**DOI:** 10.1038/s41598-018-32888-z

**Published:** 2018-10-10

**Authors:** Akiko Suzuki, Ryohei Minamide, Junichi Iwata

**Affiliations:** 1 Department of Diagnostic & Biomedical Sciences, The University of Texas Health Science Center at Houston (UTHealth) School of Dentistry, Houston, TX 77054 USA; 2 Center for Craniofacial Research, UTHealth School of Dentistry, Houston, TX 77054 USA; 3 MD Anderson Cancer Center UTHealth Graduate School of Biomedical Sciences, Houston, TX 77054 USA

## Abstract

Molecules involved in WNT/β-catenin signaling show spatiotemporal-specific expression and play vital roles in muscle development. Our previous study showed that WNT/β-catenin signaling promotes myoblast proliferation and differentiation through the regulation of the cyclin A2 (*Ccna2*)/cell division cycle 25C (*Cdc25c*) and Fermitin family homolog 2 (*Fermt2*) genes, respectively. However, it remains unclear how β-catenin targets different genes from stage to stage during myogenesis. Here, we show that the accessibility of β-catenin to the promoter region of its target genes is regulated by developmental stage-specific histone acetyltransferases (HATs), lysine acetyltransferase 2B (KAT2B), and cAMP-response element-binding protein (CREB)-binding protein (CBP). We found that KAT2B was specifically expressed at the myoblast proliferation stage and formed a complex with β-catenin to induce *Ccna2/Cdc25c* expression. On the other hand, CBP was specifically expressed during myoblast differentiation and formed a complex with β-catenin to induce *Fermt2* expression. Our findings indicate that β-catenin efficiently accesses to its target gene’s promoters by forming a complex with developmental stage-specific acetyltransferases during myogenesis.

## Introduction

Skeletal muscle development and regeneration are controlled through complex regulatory processes^1^. Muscle precursor cells (*aka* satellite cells in adults) start to proliferate, at which stage they are referred to as myoblasts, and subsequently differentiate into myotubes^2^. WNT/β-catenin signaling contributes to both muscle cell proliferation and differentiation^3^. Upon binding WNT ligands to a Frizzled receptor (FZD) and the low-density lipoprotein receptor-related protein 5/6 (LRP5/6), β-catenin can stabilize and translocate from the cytoplasm into the nucleus. Nuclear β-catenin forms a complex with transcriptional co-activators, such as members of the T-cell factor (TCF)/lymphoid enhancer-binding factor 1 (LEF1) family, to bind the promoter regions of target genes^4^. By contrast, without WNT ligands, a destruction complex, which consists of AXIN, adenomatous polyposis coli (APC), and the serine-threonine kinase glycogen synthase kinase-3 (GSK3β), is activated and phosphorylates β-catenin, leading to its degradation through the ubiquitin-proteasome system^5^. While WNT/β-catenin signaling plays a crucial role in both proliferation and differentiation during myogenesis^3^, it remains unclear how β-catenin controls the expression of genes for either proliferation or differentiation.

Post-translational modifications (e.g. acetylation, methylation, phosphorylation, and ubiquitination) of core histone proteins change chromatin conformation^6,7^. Among them, histone (H) acetylation at lysine (K) residues is crucial for the activation of gene transcription^8^. The bromodomain, a small protein domain of histone acetyl transferase (HAT), functions in the linking of protein complexes to acetylated nucleosomes, thereby controlling the transcription of target genes through chromatin structure changes. For example, H3K9 and H3K27 acetylation (H3K9Ac and H3K27Ac) activates promoter/enhancer regions of genes that are then transcribed^9^. Lysine acetyltransferase 2B (KAT2B) [*aka* p300/CBP-associated factor (PCAF)] is a transcriptional coactivator that works as a HAT for H3K9, and as an acetyl-lysine reader through its conserved bromodomain located at C-terminal of the HAT domain^10^. By contrast, CREB-binding protein (CBP) specifically acetylates H3K27 through the HAT and E1A binding domains^9^.

In this study, we examined the molecular mechanism of how WNT/β-catenin signaling regulates its target genes in a temporal-specific manner. We found that the β-catenin complex incorporates either KAT2B or CBP in a developmental stage-specific manner, regulating the accessibility of β-catenin to the target gene’s promoters.

## Results

### Temporal-specific gene regulation of *Ccna2*, *Cdc25c* and *Fermt2* during muscle differentiation *in vitro*

**Figure 1:**
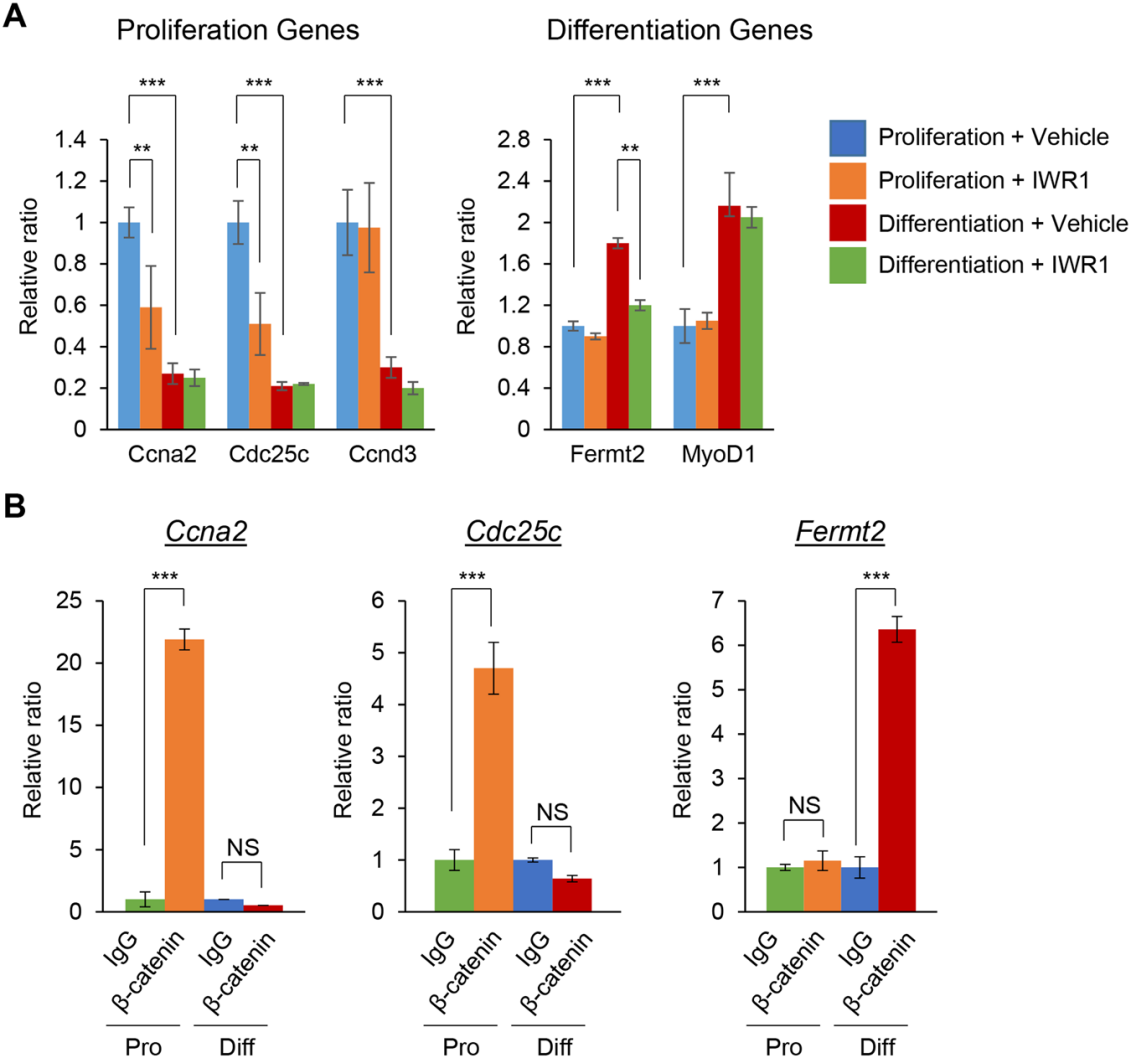
Temporal-specific binding of the β-catenin complex to the loci of target genes of WNT/β-catenin signaling. (**A**) Quantitative RT-PCR analysis of gene expression of the indicated genes in C2C12 cells treated with vehicle (control) or IWR1-endo in the proliferation and muscle differentiation stages (n = 3 each group). **p < 0.005, ***p < 0.001. (**B**) ChIP-qPCR analysis of β-catenin binding on the promoter regions of the indicated genes using β-catenin antibody. ChIP with IgG at proliferation (Pro) stage (green), α-β-catenin antibody at proliferation stage (orange), IgG at myogenic differentiation (Diff) stage (blue), and α-β-catenin antibody at myogenic differentiation stage (red) (n = 3 each group). ***p < 0.001. NS, not significant.

C2C12/myoblast precursor cells differentiate into myotubes when cultured with induction medium for muscle differentiation. Myoblast precursor cells differentiate into myoblasts within the first 24 hours after induction, and then start to fuse with each other around 48 hours post-induction. At 72 hours, the elongated multi-nucleated myotubes are detectable^3,11^. In our previous study, we found that WNT/β-catenin signaling regulates myoblast proliferation and early differentiation (myoblast fusion) through gene regulation of cyclin A2 (*Ccna2*), cell division cycle 25c (*Cdc25c*), and Fermitin family homolog 2 (*Fermt2*) in C2C12 cells^3^. However, it was still unclear how the expression of these genes was regulated by WNT/β-catenin signaling in a proliferation and differentiation stage-specific manner. The gene expression of *Ccna2* and *Cdc25c*, crucial for cell cycle progression, was much higher in the proliferation stage than in the differentiation stage and was downregulated by a WNT/β-catenin signaling inhibitor, IWR1-endo, while *Ccnd3* expression was not altered by IWR1-endo (Fig. 1A left). By contrast, gene expression of *Fermt2*, crucial for myoblast fusion, was induced in the differentiation stage compared to the proliferation stage and was downregulated by IWR1-endo, while expression of *Myod1*, *Myog* and *Myh1* was not altered by IWR1-endo (Fig. 1A right and Fig. S1). These results indicate that a gene is expressed depending on its requirement at each stage of myogenesis. To investigate the temporal-specific binding of the β-catenin complex to the promoter region of target genes, we performed chromatin immunoprecipitation (ChIP) assays with anti-β-catenin antibody for the promoter region of *Ccna2*, *Cdc25c* and *Fermt2* at both proliferation and differentiation stages. We found that β-catenin selectively accesses the locus of *Ccna2* and *Cdc25c* in the proliferation stage and the locus of *Fermt2* in the differentiation stage (Fig. 1B). These results indicate that β-catenin complex is accessible to the locus of its target genes in a developmental stage-specific manner.

### Temporal-specific expression of *Kat2b* and *Cbp* in muscle differentiation *in vitro*

**Figure 2:**
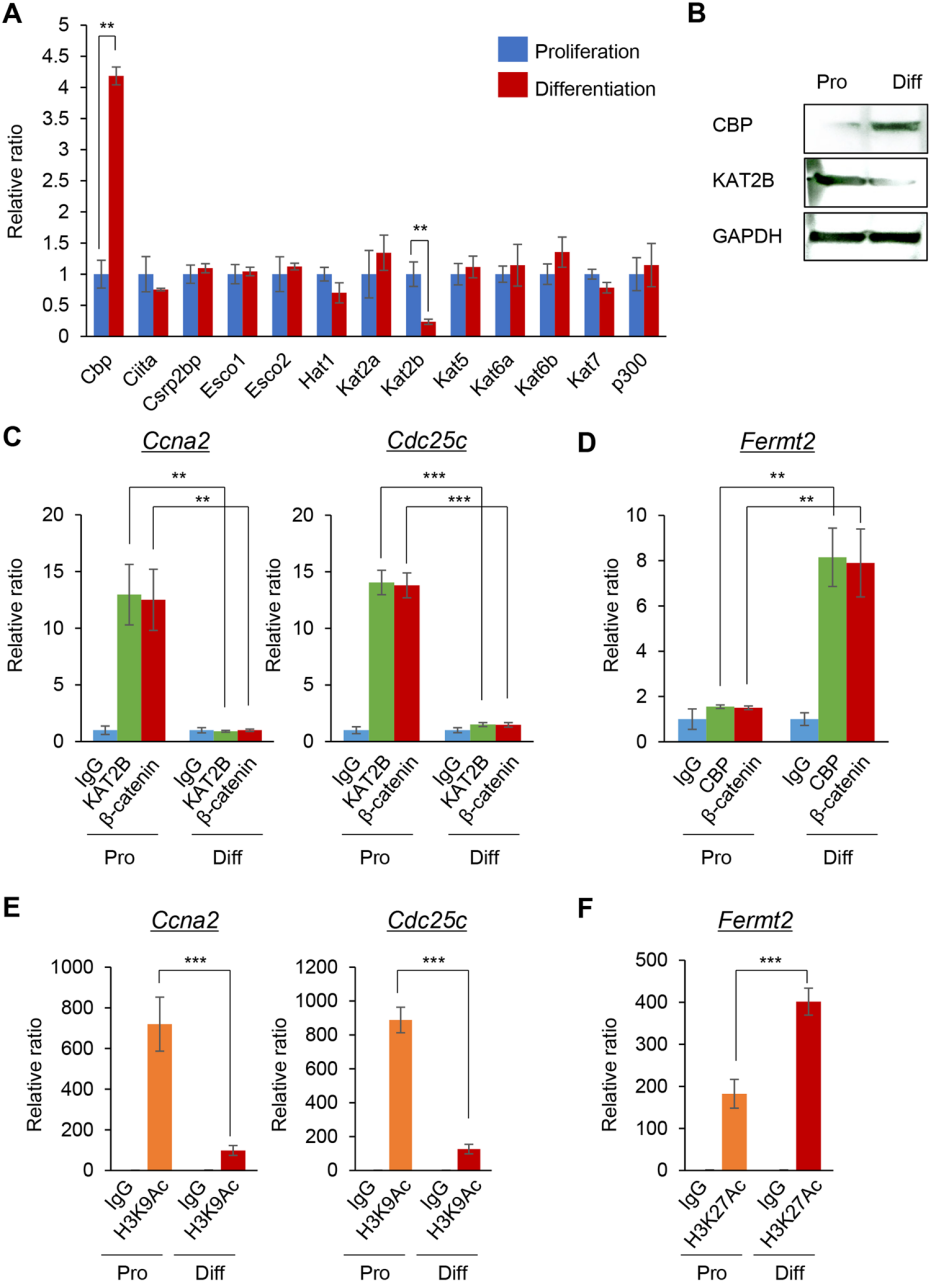
Identification of proliferation- or differentiation-specific acetyltransferases. (**A**) Quantitative RT-PCR analysis of the indicated genes in the proliferation (blue bars) and muscle differentiation (red bars) stages (n = 3 each group). **p < 0.005. (**B**) Immunoblotting analysis of KAT2B and CBP in the proliferation (Pro) and differentiation (Diff) stages. Full-length blots are presented in Supplementary Fig. 6. (**C**) ChIP-qPCR analysis of KAT2B and β-catenin binding on the promoter regions of the indicated genes. ChIP with IgG at proliferation (Pro) stage (light blue), α-KAT2B antibody at proliferation stage (light green), α-β-catenin antibody at proliferation stage (red), IgG at myogenic differentiation (Diff) stage (light blue), α-KAT2B antibody at myogenic differentiation stage (light green), and), and α-β-catenin antibody at myogenic differentiation stage (red) (n = 3 each group). **p < 0.01, ***p < 0.001. (**D**) ChIP-qPCR analysis of CBP binding on the promoter regions of *Fermt2*. ChIP with IgG at proliferation (Pro) stage (light blue), α-CBP antibody at proliferation stage (light green), α-β-catenin antibody at proliferation stage (red), IgG at myogenic differentiation (Diff) stage (light blue), α-CBP antibody at myogenic differentiation stage (light green), and α-β-catenin antibody at myogenic differentiation stage (red) (n = 3 each group). **p < 0.01. (**E**) ChIP-qPCR analysis of H3K9Ac on the promoter regions of the indicated genes. ChIP with IgG at proliferation (Pro) stage (green), α-H3K9Ac antibody at proliferation stage (orange), IgG at myogenic differentiation (Diff) stage (blue), and α-H3K9Ac antibody at myogenic differentiation stage (red) (n = 3 each group). ***p < 0.001. (**F**) ChIP-qPCR analysis of H3K27Ac on the promoter regions of *Fermt2*. ChIP with IgG at proliferation (Pro) stage (green), α-H3K27Ac antibody at proliferation stage (orange), IgG at myogenic differentiation (Diff) stage (blue), and α-H3K27Ac antibody at myogenic differentiation stage (red) (n = 3 each group). ***p < 0.001.

Promoter accessibility should depend on the chromatin state, which is modified by epigenetic factors^12^. Histone lysine acetylation is considered to be a hallmark of open chromatin^8^. To identify HATs that are differentially expressed at the myoblast proliferation and differentiation stages, we performed PCR array analyses for HATs (*Cbp*, *Ciita*, *Csrp2bp*, *Esco1*, *Esco2*, *Hat1*, *Kat2a*, *Kat2b*, *Kat5*, *Kat6a*, *Kat6b*, *Kat7* and *p300*) in C2C12 cells during cell proliferation and muscle differentiation. We found that *Kat2b* and *Cbp* were highly and specifically expressed during cell proliferation and muscle differentiation, respectively (Fig. 2A,B). To examine whether KAT2B and CBP affected the accessibility of β-catenin to the target gene promoters, we conducted ChIP assays for KAT2B and CBP in the proliferation and differentiation stages. KAT2B selectively bound to the *Ccna2* and *Cdc25c* promoters during the cell proliferation stage, but not in the differentiation stage (Fig. 2C). As expected, KAT2B did not bind to the *Fermt2* promoter in each stage of myogenesis (Fig. S2A). On the other hand, CBP selectively bound to the *Fermt2* promoter in the differentiation stage, but not in the cell proliferation stage (Fig. 2D). CBP did not bind to the *Ccna2* and *Cdc25c* promoters in each stage of myogenesis (Fig. S2B). We also found that β-catenin occupancy was similar to that of each acetyltransferase at each stage of the cells’ differentiation process. This suggests that β-catenin has a role in different HAT complexes at different stages (Fig. 2C,D). Because KAT2B and CBP are H3K9 and H3K27 acetyltransferases, respectively^9,10^, we further examined H3K9 acetylation (H3K9Ac) and H3K27Ac in the promoter regions of *Ccna2*, *Cdc25c* and *Fermt2*. H3K9Ac was markedly increased in the proliferation stage on the *Ccna2* and *Cdc25c* promoters, compared to the muscle differentiation stage (Fig. 2E). By contrast, H3K27Ac was increased on the *Fermt2* promoter in the muscle differentiation stage compared to the cell proliferation stage (Fig. 2F). We confirmed that H3K27Ac in the *Ccna2* and *Cdc25c* promoters, and H3K9Ac in the *Fermt2* promoter, were not altered at each stage of myogenesis (Fig. S2C,D). Taken together, our results indicate that KAT2B and CBP regulate the chromatin state of the *Ccna2* and *Cdc25c* promoters and the *Fermt2* promoter in cell proliferation stage and differentiation stage, respectively.

**Figure 3:**
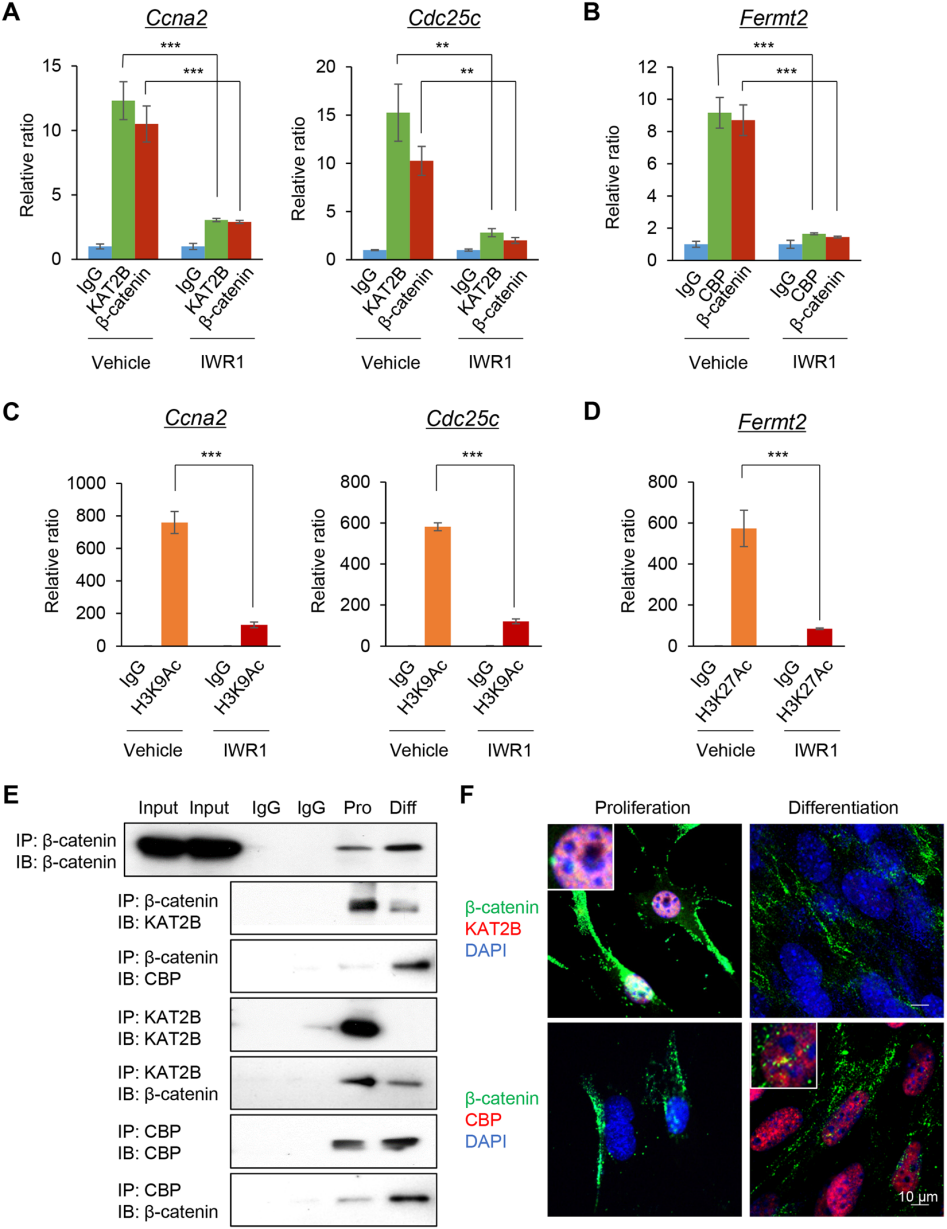
HATs and β-catenin coordinately access the loci of target genes of WNT/β-catenin signaling. (**A**) ChIP-qPCR analysis of KAT2B and β-catenin binding on the *Ccna2* and *Cdc25c* promoter regions with/without 80 μM IWR1-endo treatment for 24 hours at the proliferation stage. ChIP with IgG without IWR1-endo (vehicle; light blue), IgG with IWR1-endo (light blue), α-KAT2B antibody without IWR1-endo (vehicle; light green), α-KAT2B antibody with IWR1-endo (light green), α-β-catenin antibody without IWR1-endo (vehicle; red), and α-β-catenin antibody with IWR1-endo (red) (n = 3 each group). **p < 0.01, ***p < 0.001. (**B**) ChIP-qPCR analysis of CBP acetylation on the *Fermt2* promoter regions with/without 1 μM IWR1-endo for 48 hours at the differentiation stage. ChIP with IgG without IWR1-endo (vehicle; light blue), IgG with IWR1-endo (light blue), α-CBP antibody without IWR1-endo (vehicle; light green), α-CBP antibody with IWR1-endo (light green), α-β-catenin antibody without IWR1-endo (vehicle; red), and α-β-catenin antibody with IWR1-endo (red) (n = 3 each group). ***p < 0.001. (**C**) ChIP-qPCR analysis of H3K9Ac on the *Ccna2* and *Cdc25c* promoter regions with/without 80 μM IWR1-endo for 24 hours at the proliferation stage. ChIP with IgG without IWR1-endo (vehicle; green), IgG with IWR1-endo (blue), α-H3K9Ac antibody without IWR1-endo (vehicle; orange), and α-H3K9Ac antibody with IWR1-endo (red) (n = 3 each group). ***p < 0.001. (**D**) ChIP-qPCR analysis of H3K27 acetylation on the *Fermt2* promoter regions with/without 1 μM IWR1-endo for 48 hours at the differentiation stage. ChIP with IgG without IWR1-endo (vehicle; green), IgG with IWR1-endo (blue), α-H3K27Ac antibody without IWR1-endo (vehicle; orange), and α-H3K27Ac antibody with IWR1-endo (red) (n = 3 each group). ***p < 0.001. (**E**) IP-immunoblotting (IB) analysis with the indicated antibodies after IP for the indicated antibodies using nuclear extracts from either proliferating (Pro) or differentiated (Diff) C2C12 cells. 20% input and IgG control from either proliferating or differentiated C2C12 cells. Full-length blots are presented in Supplementary Fig. 6. (**F**) Immunofluorescence analyses for β-catenin (green), KAT2B (red), and CBP (red) during cell proliferation and differentiation. DAPI (blue) was used for nuclear staining. Inserts show higher magnification from the images. Bars, 10 µm.

Next, to assess whether KAT2B and CBP were correlated with WNT/β-catenin signaling activity, we treated C2C12 cells with IWR1-endo. IWR1-endo inhibited the binding of KAT2B to the *Ccna2* and *Cdc25c* promoters during the cell proliferation stage (Fig. 3A). On the other hand, IWR1-endo inhibited the binding of CBP to the *Fermt2* promoter in the differentiation stage (Fig. 3B). The occupancy of β-catenin in these promoters was similar to that of KAT2B and CBP with or without IWR1-endo treatment (Fig. 3A,B). In addition, we confirmed that IWR1-endo inhibited H3K9Ac on the *Ccna2* and *Cdc25c* promoters during cell proliferation and H3K27Ac on the *Fermt2* promoter during muscle differentiation (Fig. 3C,D). The knockdown of *Ctnnb1* showed outcomes similar to those of IWR1-endo treatment, indicating that IWR1-endo specifically inhibits WNT/β-catenin signaling (Fig. S3). These results indicate that WNT/β-catenin signaling is crucial for KAT2B-mediated H3K9Ac and CBP-mediated H3K27Ac in the promoter of *Ccna2*/*Cdc25c* and *Fermt2*, respectively.

### Temporal-specific incorporation of KAT2B and CBP into the β-catenin complex during muscle differentiation *in vitro*

We hypothesized that β-catenin forms a complex with HATs in order to access efficiently the target gene’s promoters in a proliferation and differentiation stage-specific manner. To investigate the interaction between HATs and β-catenin, we conducted co-immunoprecipitation (co-IP) at the proliferation and differentiation stages. The interaction between β-catenin and KAT2B was increased in the proliferation stage. By contrast, the interaction between β-catenin and CBP was increased in the differentiation stage (Fig. 3E). Furthermore, we examined the co-localization of β-catenin with KAT2B or CBP by immunofluorescence analyses. KAT2B was highly expressed in the proliferation stage and co-localized with β-catenin in the nuclei in 74.7% of the cells (n = 87). On the other hand, CBP was highly expressed in the differentiation stage and co-localized with β-catenin in the nuclei in 60.9% of the cells (n = 46) (Fig. 3F). Taken together, our results indicate that β-catenin forms a complex with stage-specific HATs in the nuclei so that WNT/β-catenin signaling controls gene expression on demand.

### Role of *Kat2b* and *Cbp* in the cell proliferation and muscle differentiation stages

**Figure 4:**
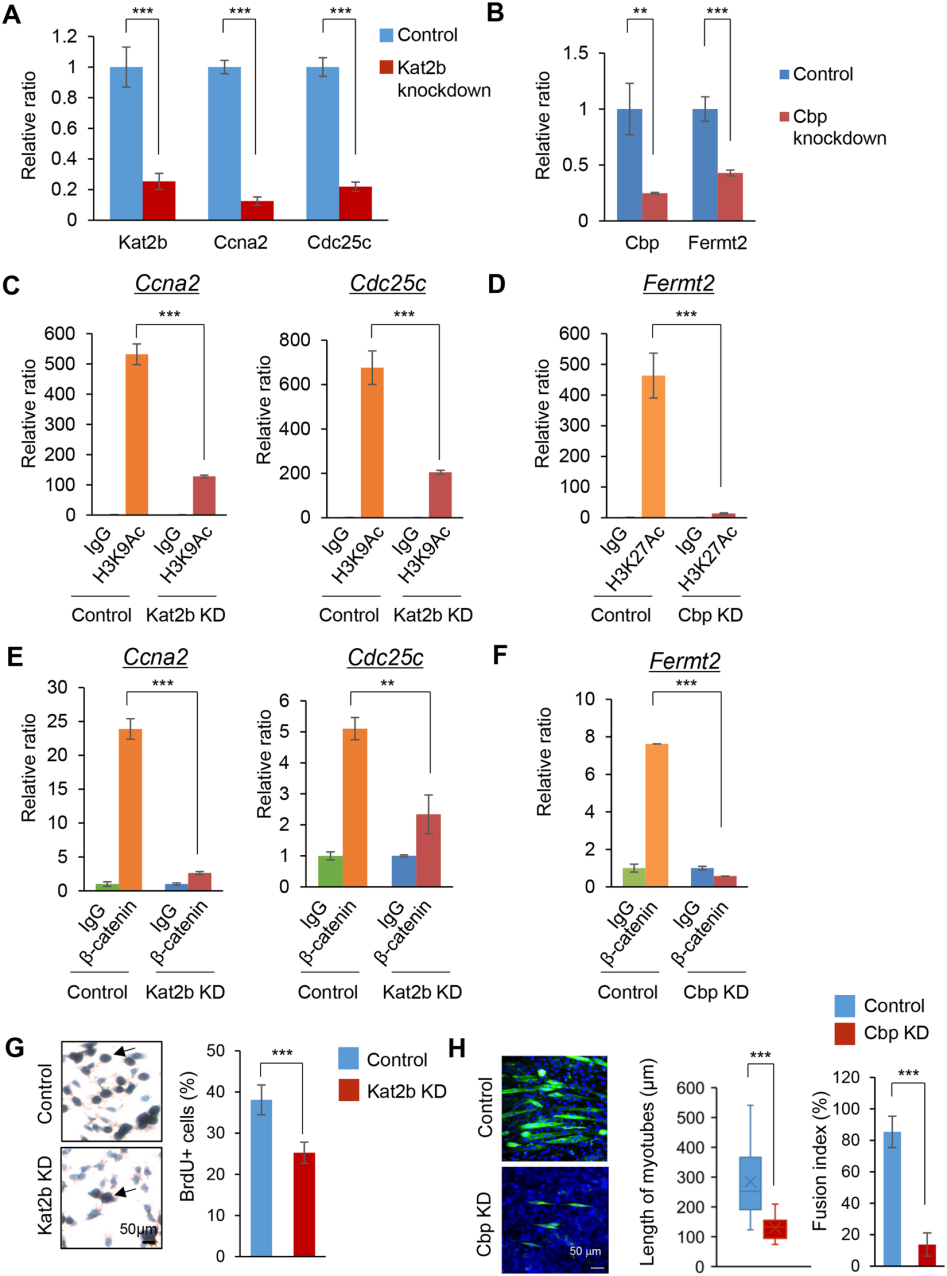
HATs and β-catenin coordinately regulate cell proliferation and differentiation through the regulation of *Ccna2*, *Cdc25c* and *Fermt2*. (**A**) Quantitative RT-PCR analysis of indicated genes after siRNA knockdown of control (blue) or *Kat2b* (red) (n = 3 each group). ***p < 0.001. (**B**) Quantitative RT-PCR analysis of indicated genes after siRNA knockdown of control (blue) or *Cbp* (red) (n = 3 each group). **p < 0.01, ***p < 0.001. (**C**) ChIP-qPCR analysis of H3K9Ac on the *Ccna2* and *Cdc25c* promoter regions with *Kat2b* siRNA knockdown for 24 hours at the proliferation stage. ChIP with IgG with control siRNA (green), IgG with *Kat2b* siRNA knockdown (blue), α-H3K9Ac antibody with control siRNA (orange), and α-H3K9Ac antibody with *Kat2b* siRNA knockdown (red) (n = 3 each group). ***p < 0.001. (**D**) ChIP-qPCR analysis of H3K27Ac on the *Fermt2* promoter region after *Cbp* siRNA knockdown for 24 hours at the proliferation stage. ChIP with IgG with control siRNA (green), IgG with *Cbp* siRNA knockdown (blue), α-H3K27Ac antibody with control siRNA (orange), and α-H3K27Ac antibody with *Cbp* siRNA knockdown (red) (n = 3 each group). ***p < 0.001. (**E**) ChIP-qPCR analysis of β-catenin binding on the *Ccna2* and *Cdc25c* promoter regions following *Kat2b* siRNA knockdown for 24 hours at the proliferation stage. ChIP with IgG with control siRNA (green), IgG with *Kat2b* siRNA knockdown (blue), α-β-catenin antibody with control siRNA (orange), and α-β-catenin antibody with *Kat2b* siRNA knockdown (red) (n = 3 each group). **p < 0.01, ***p < 0.001. (**F**) ChIP-qPCR analysis of β-catenin binding on the *Fermt2* promoter region following *Cbp* siRNA knockdown for 24 hours at the proliferation stage. ChIP with IgG with control siRNA (green), IgG with *Cbp* siRNA knockdown (blue), α-β-catenin antibody with control siRNA (orange), and α-β-catenin antibody with *Cbp* siRNA knockdown (red) (n = 3 each group). ***p < 0.001. (**G**) BrdU staining in C2C12 cells after control or *Kat2b* siRNA knockdown (KD) for 24 h. Nuclei are counterstained with hematoxylin. Arrows indicate BrdU-positive cells. Scale bar, 50 µm. Graph shows the quantification of BrdU-positive cells. ***p < 0.001. (**H**) Immunocytochemical analysis of MYH (green) in differentiated C2C12 cells with control or *Cbp* siRNA knockdown (KD). Scale bar, 50 µm. Graph shows the quantification of the length of myotubes (middle) and fusion index (right). ***p < 0.001.

To examine the functional significance of KAT2B and CBP in the regulation of *Ccna2*, *Cdc25c* and *Fermt2*, we performed knockdown experiments for *Ccna2*, *Cdc25c* and *Fermt2* in the cell proliferation and differentiation stages. *Kat2b* knockdown decreased the expression of *Ccna2* and *Cdc25c* in the cell proliferation stage (Fig. 4A). On the other hand, *Cbp* knockdown decreased the expression of *Fermt2* in the differentiation stage (Fig. 4B). Knockdown of *Kat2b* and *Cbp* decreased H3K9Ac and H3K27Ac, respectively (Fig. 4C,D). In addition, knockdown of *Kat2b* and *Cbp* decreased the binding of β-catenin to the *Ccna2* and *Cdc25c* promoters and the *Fermt2* promoter in the cell proliferation stage and the differentiation stage, respectively (Fig. 4E,F). To examine the role of *Kat2b* and *Cbp* in cell proliferation and differentiation, we conducted functional assays for cell proliferation and muscle differentiation after knockdown of *Kat2b* and *Cbp*. *Kat2b* knockdown significantly decreased cell proliferation (Fig. 4G). Because the reduction in proliferation caused by *Kat2b* knockdown was not drastic, we checked whether KAT2B-mediated acetylation had other roles in the proliferating muscle cells, such as myogenic lineage determination and survival. We examined whether cell fate was switched by *Kat2b* knockdown in the proliferating cells by performing qRT-PCR for *Myog*, *Cdkn1a/p21*, and *Myh1* and found that there was no difference in the expression level of these genes between the control and knockdown groups (Fig. S4A). We also carried out immunofluorescence staining for MyoD to check the myogenic status of *Kat2b* knockdown cells in the proliferation stage (Fig. S4B). The percentage of MyoD-positive cells did not change after *Kat2b* knockdown. Taken together, these results suggest that *Kat2b* knockdown inhibited proliferation, but did not induce myogenic cell survival, and differentiation was not affected by the expression level of *Kat2b*. On the other hand, *Cbp* knockdown resulted in muscle differentiation defects as seen in the reduction in the length of myotubes and the fusion index (Fig. 4H). To test whether CBP levels regulate fate switching, we carried out quantitative RT-PCR for *Myod1* and *Myog* (Fig. S4C) and immunofluorescence staining for BrdU (Fig. S4D). There was no change in these results after *Cbp* knockdown, indicating that *Cbp* knockdown did not affect the cell fate. Furthermore, in the proliferation stage, a combined treatment of *Cbp* overexpression and *Kat2b* knockdown failed to induce the expression of genes related to myogenic differentiation (*MyoD1*, *Myog*, and *Myh1*) compared to controls (Fig. S5A). Similarly, in the differentiation stage, another combined treatment of *Kat2b* overexpression and *Cbp* knockdown failed to alter the expression of genes related to cell proliferation and differentiation (Fig. S5B). These results suggest that WNT/β-catenin-mediated specific switch of HATs in the proliferation and differentiation stages is not reversible. Taken together, our results indicate that KAT2B and CBP regulate myoblast proliferation and differentiation through the regulation of the expressions of *Ccna2*, *Cdc25c* and *Fermt2*.

## Discussion

Altered WNT/β-catenin signaling has been found in multiple malformations and syndromes, including muscle disorders in humans^13–15^. For example, in patients with muscular defects, such as myopathy and atrophy, WNT/β-catenin signaling is altered due to genetic and epigenetic factors^16^. Our previous study indicates that WNT/β-catenin signaling contributes to both myoblast proliferation and differentiation through the regulation of proliferation and differentiation stage-specific genes^3^. However, it has been unclear how WNT/β-catenin can specifically induce downstream target genes at each stage of muscle development. Epigenetic factors along with cell signaling activation have been implicated in the regulation of stage-specific gene expression^17,18^. During embryogenesis, chromatin conformation dramatically changes at each stage of development, and chromatin is opened at specific regions to transcribe target genes^17,18^. In this study, we found that KAT2B and CBP were specifically expressed during the stage of myoblast proliferation and differentiation, respectively. In addition, the induction of WNT/β-catenin target genes was correlated with the acetylation of the WNT/β-catenin response elements on the promoters of target genes. There would be an effective mechanism through which cell cycle activators are induced during proliferation and suppressed during differentiation, and the same for molecules involved in muscle differentiation. Our results indicate that β-catenin directly interacts with a specific HAT because it opens a specific chromatin domain. In this process, β-catenin forms a complex with epigenetic factors that allow efficient recognition of the loci of target genes. Because this is an efficient way to induce the target genes, it may be conserved in other cell signaling pathways. For example, a previous study indicates that KAT2B induces H3K9Ac on the *Gli1* target gene promoters in order to induce cell proliferation in cancer cells^19^. Because hedgehog signaling plays an important role in myogenesis^20^, H3K9Ac may be a downstream of not only WNT/β-catenin signaling, but also hedgehog signaling pathway in myogenesis. Thus, epigenetic factors are expressed in a temporal-specific manner and involved in switching cell fate from cell proliferation to differentiation.

A number of HATs have been identified in mammals, suggesting that each acetyltransferase may have a different function, expression pattern, and regulation^21^. Previous studies indicate that β-catenin can bind to various molecules including acetyltransferases^22,23^. For example, in PC12 cells, CBP/p300 interacts with β-catenin to promote neuronal differentiation^24^. In C2C12 muscle cells, microinjection of CBP/p300 antibodies inhibits muscle differentiation^25,26^, indicating that CBP/p300 is crucial for muscle differentiation. In addition, histone acetylation by CBP/p300 regulates neuronal progenitor cell fate by switching from self-renewal to differentiation^27^. This suggests that contribution of CBP to differentiation may be widely conserved in other cell types. In this study, we found that KAT2B and CBP could form a complex with β-catenin at different stages of muscle development *in vitro*. Further studies will determine whether there is a specific domain for the interactions between β-catenin and KAT2B or CBP. In addition, recent studies show that acetylation of β-catenin itself by either KAT2B or CBP affects the stability of β-catenin^28–30^. This suggests that histone and non-histone acetylation may work as a positive feedback loop in order to induce WNT/β-catenin downstream targets. During proliferation, although β-catenin and CBP occupancies were very low on the *Fermt2* promoter, H3K27Ac occupancy was significantly enriched. Previous studies suggest that low levels of CBP are sufficient for H3K27Ac occupancy on various promoters^31,32^. Low levels of CBP might be sufficient for H3K27Ac occupancy on *Fermt2* promoter during proliferation as well.

While we focused on DNA acetylation in this study, other epigenetic factors potentially modulate gene expression in a spatiotemporal-specific manner during myogenesis. For example, several microRNAs (e.g., miR-23a, miR-133, miR-206) are specifically expressed in skeletal muscle and differentially regulate the gene expression of myogenic factors^33–35^. DNA methylation status is altered in muscle development and diseases^36,37^. Thus, various epigenetic factors may also modulate the WNT/β-catenin signaling activity during skeletal muscle development and diseases.

In summary, our results indicate that epigenetic factors play crucial roles in the regulation of WNT/β-catenin signaling target gene by directly interacting with β-catenin. Our findings on the epigenetic regulation of WNT/β-catenin signaling offer several intriguing possibilities into the potential for therapeutic interventions to stimulate effective skeletal muscle regeneration following muscle trauma or atrophy.

## Methods

### Cell culture

C2C12 cells, a murine skeletal muscle cell line, were obtained from the American Type Culture Collection (ATCC; CRL-1772). C2C12 cells were cultured in growth medium [Dulbecco’s Modified Eagle’s Medium (DMEM) supplemented with 10% fetal bovine serum, penicillin, streptomycin, 2 mM L-glutamate, 1 mM sodium pyruvate, and nonessential amino acids] for 24 hours at 37 °C and 5% CO_2_ in a humidified incubator, as previously described^3^. Myogenic differentiation was induced with muscle differentiation medium [DMEM supplemented with 2% horse serum, 2 mM L-glutamate, and penicillin/streptomycin] for 48 hours with/without IWR1-endo (Tocris Bioscience, Bristol, UK), a WNT/β-catenin signaling inhibitor, at the indicated concentration, as previously described^3^. The small interfering RNA (siRNA) knockdown for *Kat2b*, *Cbp* and *Ctnnb1* (Santa Cruz) was performed as described previously^3^. Bromodeoxyuridine (BrdU) incorporation assays were performed using cells treated with control or *Kat2b* siRNA knockdown for 24 hours and control or *Cbp* siRNA knockdown for 48 hours. Incorporated BrdU was stained with a rat monoclonal antibody against BrdU (Abcam), as described previously^3^. A total of 10 fields, randomly selected from three independent experiments, were used for the quantification of BrdU-positive cells. The antibodies used in this study are shown in Supplementary Table S1.

### Quantitative RT-PCR

For the cell proliferation stage, total RNAs isolated from C2C12 cells, treated with IWR1-endo or control vehicle for 24 hours (n = 3 per group) and *Kat2b* siRNA or control siRNA for 24 hours (n = 3 per group), were extracted with a QIAshredder and RNeasy mini extraction kit (QIAGEN), as described previously^38,39^. For the myoblast differentiation stage, total RNAs isolated from C2C12 cells cultured under differentiation medium with 1 µM IWR1-endo or control vehicle for two days (n = 3 per group) and *Cbp* siRNA or control siRNA for two days (n = 3 per group) were dissected with the QIAshredder and RNeasy mini extraction kit (QIAGEN), as described previously^40^. The PCR primer pairs used for further specific analysis are shown in Supplemental Table S2.

### ChIP assay

Cell extracts were incubated with either active β-catenin, H3K9Ac, or H3K27Ac antibody (Cell Signaling Technology) overnight at 4 °C, followed by precipitation with magnetic beads. Washing and elution of the immune complexes, as well as precipitation of DNA, were performed according to standard procedures, as described previously^40,41^. The putative LEF1 target sites of the genes in the immune complexes were detected by PCR using the following primers: *Ccna2* gene, 5′-TGGTGTTGCAGATCTACCGT-3′ and 5′-TCTGCTAACAAAATGGCAATGC-3′ (−2364 to −2154); *Cdc25c* gene, 5′-TTCATCGGTCTCAGCTTCCC-3′ and 5′-AGTCACCACTGAGCCTTGTC-3′ (−3164 to −3029); and *Fermt2* gene, 5′-TAGAGGTTCTAGCGGGGGTT-3′ and 5′-TTCAGGCCTTGGCTTTGAGT-3′ (−3138 to −2949). The positions of the PCR fragments correspond to NCBI mouse genome Build 38 (mm10).

### Immunoprecipitation and immunoblotting

Nuclear extracts were prepared for IP using a Nuclear Extraction Kit (Thermo Scientific). IP assays were performed as described previously^41^. Mouse monoclonal antibodies against β-catenin (Abcam), KAT2B (Santa Cruz) and CBP (Abcam) were used for IP assays. Immunoblots were performed using rabbit polyclonal antibodies against active β-catenin, KAT2B, and CBP (Cell Signaling Technology), as described previously^42^.

### Immunofluorescence analysis

Immunofluorescence analysis was performed, as described previously^3^, using the following antibodies: mouse monoclonal antibody against active β-catenin (Millipore), rabbit polyclonal antibodies against KAT2B (Cell Signaling Technology) and CBP (Cell Signaling Technology) and mouse monoclonal antibodies against myosin heavy chain (MYH) (Sigam-Aldrich) and MyoD1 (Thermofisher). Confocal images were obtained with a confocal microscope (C2, Nikon). Fluorescent images of MYH immunostaining were captured by an inverted fluorescence microscope (IX73, Olympus). A total of 10 fields, randomly selected from three independent experiments, were used for the quantification of the length of myotubes and fusion index.

### Statistical analysis

Two-tailed student’s *t* tests were applied for statistical analysis. A *p* value ≤ 0.05 was considered statistically significant. For all graphs, data are represented as mean ± standard deviation (SD).

## Electronic supplementary material

Supplemental Information
