# Supplementary material for: The role of acetyltransferases for the temporal-specific accessibility of β-catenin to the myogenic gene locus
Source: Sci Rep. 2018 Oct 10;8:15057. doi: 10.1038/s41598-018-32888-z (PMC6180044; doi:10.1038/s41598-018-32888-z)

Supplementary Information

The role of acetyltransferases for the temporal-specific accessibility of β-catenin to the myogenic gene locus

Akiko Suzuki[1,2], **Ryohei Minamide**[1,2], **and Junichi Iwata**[1,2,3,*]

[1]Department of Diagnostic & Biomedical Sciences, The University of Texas Health Science Center at Houston (UTHealth) School of Dentistry, Houston, TX 77054, USA

[2]Center for Craniofacial Research, UTHealth School of Dentistry, Houston, TX 77054, USA

[3]MD Anderson Cancer Center UTHealth Graduate School of Biomedical Sciences, Houston, TX 77054, USA

*Corresponding author: Junichi.Iwata@uth.tmc.edu

Supplementary Figure 1. WNT/β-catenin signaling does not regulate gene expression of *Myog* and *Myh1* during muscle differentiation. Quantitative RT-PCR analysis of gene expression of the indicated genes with vehicle (control) or IWR1-endo in the proliferation and muscle differentiation stages (n=3 each group).

Supplementary Figure 2. Failure of CBP binding to the *Ccna2* and *Cdc25c* promoter and KAT2B binding to the *Fermt2* promoter. (**A**) ChIP-qPCR analysis of CBP binding on the *Fermt2* promoter regions. ChIP with IgG at proliferation (Pro) stage (green), α-CBP antibody at proliferation stage (orange), IgG at myogenic differentiation (Diff) stage (blue), and α-CBP antibody at myogenic differentiation stage (red) (n=3 each group). NS, not significant. (**B**) ChIP-qPCR analysis of KAT2B binding on the promoter regions of *Ccna2* and *Cdc25c*. ChIP with IgG at proliferation (Pro) stage (green), α-KAT2B antibody at proliferation stage (orange), IgG at myogenic differentiation (Diff) stage (blue), and α-KAT2B antibody at myogenic differentiation stage (red) (n=3 each group). NS, not significant. (**C**) ChIP-qPCR analysis of H3K27Ac on the *Fermt2* promoter regions. ChIP with IgG at proliferation (Pro) stage (green), α-H3K27Ac antibody at proliferation stage (orange), IgG at myogenic differentiation (Diff) stage (blue), and α-H3K27Ac antibody at myogenic differentiation stage (red) (n=3 each group). NS, not significant. (**D**) ChIP-qPCR analysis of H3K9Ac on the promoter regions of *Ccna2* and *Cdc25c*. ChIP with IgG at proliferation (Pro) stage (green), α-H3K9Ac antibody at proliferation stage (orange), IgG at myogenic differentiation (Diff) stage (blue), and α-H3K9Ac antibody at myogenic differentiation stage (red) (n=3 each group). NS, not significant.

Supplementary Figure 3. HATs and β-catenin coordinately access the loci of target genes of WNT/β-catenin signaling. (**A**) ChIP-qPCR analysis of KAT2B and β-catenin binding on the *Ccna2* and *Cdc25c* promoter regions with control or *Ctnnb1* siRNA for 24 hours at the proliferation stage. ChIP with IgG with control siRNA (light blue), IgG with *Ctnnb1* siRNA (light blue), α-KAT2B antibody with control siRNA (light green), and α-KAT2B antibody with *Ctnnb1* siRNA (light green), α-β-catenin antibody with control siRNA (red), and α-β-catenin antibody with *Ctnnb1* siRNA (red) (n=3 each group). ***, p<0.001. (**B**) ChIP-qPCR analysis of CBP acetylation on the *Fermt2* promoter regions with control or *Ctnnb1* siRNA for 48 hours at the differentiation stage. ChIP with IgG with control siRNA (light blue), IgG with *Ctnnb1* siRNA (light blue), α-KAT2B antibody with control siRNA (light green), and α-KAT2B antibody with *Ctnnb1* siRNA (light green), α-β-catenin antibody with control siRNA (red), and α-β-catenin antibody with *Ctnnb1* siRNA (red) (n=3 each group). ***, p<0.001. (**C**) ChIP-qPCR analysis of H3K9Ac on the *Ccna2* and *Cdc25c* promoter regions with control or *Ctnnb1* siRNA for 24 hours at the proliferation stage. ChIP with IgG with control siRNA (green), IgG with *Ctnnb1* siRNA (blue), α-H3K9Ac antibody with control siRNA (orange), and α-H3K9Ac antibody with *Ctnnb1* siRNA (red) (n=3 each group). ***, p<0.001. (**D**) ChIP-qPCR analysis of H3K27 acetylation on the *Fermt2* promoter regions with control or *Ctnnb1* siRNA for 48 hours at the differentiation stage. ChIP with IgG with control siRNA (green), IgG with *Ctnnb1* siRNA (blue), α-H3K27Ac antibody with control siRNA (orange), and α-H3K27Ac antibody with *Ctnnb1* siRNA (red) (n=3 each group). ***, p<0.001.

Supplementary Figure 4. The expression levels of *Kat2b* and *Cbp* have no effect on fate determination in C2C12 cells. (**A**) Quantitative RT-PCR analysis of the indicated genes after treatment with control or *Kat2b* siRNA in the proliferation stage (n=3 each group). (**B**) The

percentage of MyoD-positive cells after treatment with control or *Kat2b* siRNA in the proliferation stage (n=3 each group). (**C**) Quantitative RT-PCR analysis of the indicated genes after treatment with control or *Cbp* siRNA in the muscle differentiation stages (n=3 each group). (**D**) BrdU staining after treatment with control or *Cbp* siRNA in the muscle differentiation stages. The graph shows quantification of BrdU-positive cells after treatment with control or *Cbp* siRNA in the muscle differentiation stages (n=3 each group).

Supplementary Figure 5. The cell fate is not reversible with the control siRNAs of *Kat2b* and *Cbp* in C2C12 cells. (**A**) Quantitative RT-PCR analysis of the indicated genes after treatment with control siRNA and control vector (blue bars) or *Kat2b* siRNA and *Cbp* overexpression (red) in the proliferation stage (n=3 each group). NS, not significant. Overexpression vectors for *Cbp* (addgene, #32908) and *Kat2b* (GenScript, #OMu11684) were transfected with Lipofectamine 3000 (Thermofisher), following manufacturer's protocol. (**B**) Quantitative RT-PCR analysis of the indicated genes after treatment with control siRNA and control vector (blue bars) or *Cbp* siRNA and *Kat2b* overexpression (red bars) in the muscle differentiation stages (n=3 each group). NS, not significant.

Supplementary Figure 6. Full-length blots.

Supplementary Table S1. Antibodies used in this study.

Antibody name	Provider	Catalog number	Application	Concentration
Acetyl-Histone H3 (Lys9)	Cell Signaling Technology	9649	ChIP	1:50
Acetyl-Histone H3 (Lys27)	Cell Signaling Technology	8173	ChIP	1:100
BrdU	Abcam	ab6321	IF, IHC	1:1000
β-catenin	Abcam	ab16051	IP	5 μg
			IF	1:100
CBP	Cell Signaling Technology	7389	ChIP	1:50
			IF	1:100
			WB	1:1000
CBP	Abcam	ab2832	IP	5 μg
GAPDH	Millipore	MAB374	WB	1:5000
KAT2B	Cell Signaling Technology	3378	ChIP	1:25
			IF	1:100
			WB	1:1000
KAT2B	Santa Cruz Biotechnology	sc-13124	IP	5 μg
Myh1	Sigma-Aldrich	MY-32	IF	1:1000
MyoD1	ThermoFisher	MA5-12902	IF	1:10
non-phosphorylated active β-catenin	Cell Signaling Technology	8814	ChIP	1:100
			IP	1:100
			IF	1:100
			WB	1:1000

Supplementary Table S2. Primer pairs used in this study.

Gene	Forward primer	Reverse primer
Cbp	5’-GGCTTCTCCGCGAATGACAA-3’	5’-GTTTGGACGCAGCATCTGGA-3’
Ccna2	5’-TTGCTCCTCTTAAGGACCTTCC-3’	5’-CATTTAACCTCCATTTCCCTAAGGT-3’
Ccnd3	5’-ATACTGGATGCTGGGGTGTG-3’	5’-TGCAGAATCAAGGCCAGGAA-3’
Cdc25c	5’-TGACGTCTATAGCCCCACC-3’	5’-TAAGCGGAGAGGCAGACATC-3’
Cdkn1a/p21	5’-TAAGGACGTCCCACTTTGCC-3’	5’-CGTCTCCGTGACGAAGTCAA-3’
Ciita	5’-TGCGTGTGATGGATGTCCAG-3’	5’-CCAAAGGGGATAGTGGGTGTC-3’
Csrp2bp	5’-CCGTCAAGTTCATAAGCCGAG-3’	5’-TCAGGGACGAGAAGTCGATCA-3’
Esco1	5’-ATGAGTCTGAATTTGGAATGCGA-3’	5’-GTCTTTGTGATCTGCGGTGAA-3’
Esco2	5’-GACCTATAAGCCAGTTGTGGAC-3’	5’-TCCGCCTTGGAGTGTAACTTG-3’
Fermt2	5’-GATCACTTTGGAAGGCGGGA-3’	5’-GCGCGTACTGCTTCTCGTTA-3’
Hat1	5’-AAGTGTAACACCAACACAGCA-3’	5’- CGAAAGCAGTTTCATCATCCCC-3’
Kat2a	5’-AAGGCCAATGAAACCTGCAAG-3’	5’- CTCACAGCTACGGCACAACTC-3’
Kat2b	5’-CGGATCGCCGTGAAGAAGG-3’	5’-CATTGCATTTACAGGACTCCTCT-3’
Kat5	5’-TCCCGGTCCAGATCACACTC-3’	5’- ACCTTCCGTTTCGTTGAGCG-3’
Kat6a	5’-CCTCGTGCATTGGCTGTTC-3’	5’-TCATGGCATTCAAGGTGTTCAT-3’
Kat6b	5’-AGAAGAAAAGGGGTCGTAAACG-3’	5’-GTGGGAATGCTTTCCTCAGAA-3’
Kat7	5’-ATGCCGCGAAGGAAGAGAAAT-3’	5’- TCTTGGGAACTCTGGCTTAGC-3’
Myf5	5’-CGGCATGCCTGAATGTAACAG-3’	5’-GCTGGACAAGCAATCCAAGC-3’
Myod1	5’-TGCTCTGATGGCATGATGGATT-3’	5’-AGATGCGCTCCACTGTGCTG-3’
Myog	5’-TCCCAACCCAGGAGCTCATT-3’	5’-AGTTGGGCATGGTTTCGTCT-3’
Myh1	5’-CGGTCGAAGTTGCATCCCTA-3’	5’-TTACAGTAGTTCCGCCTTCGG-3’
p300	5’-TTCAGCCAAGCGGCCTAAA-3’	5’-CGCCACCATTGGTTAGTCCC-3’
Gapdh	5’-AACTTTGGCATTTGGAAGG-3’	5’-ACACATTGGGGGTAGGAACA-3’

Fig. S1. Suzuki et al.

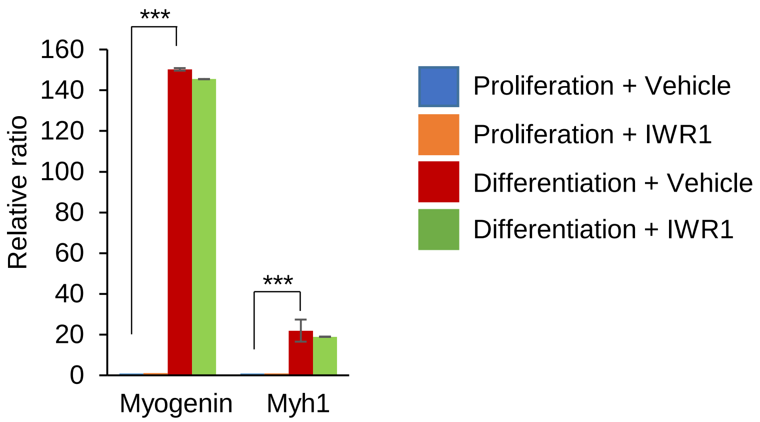

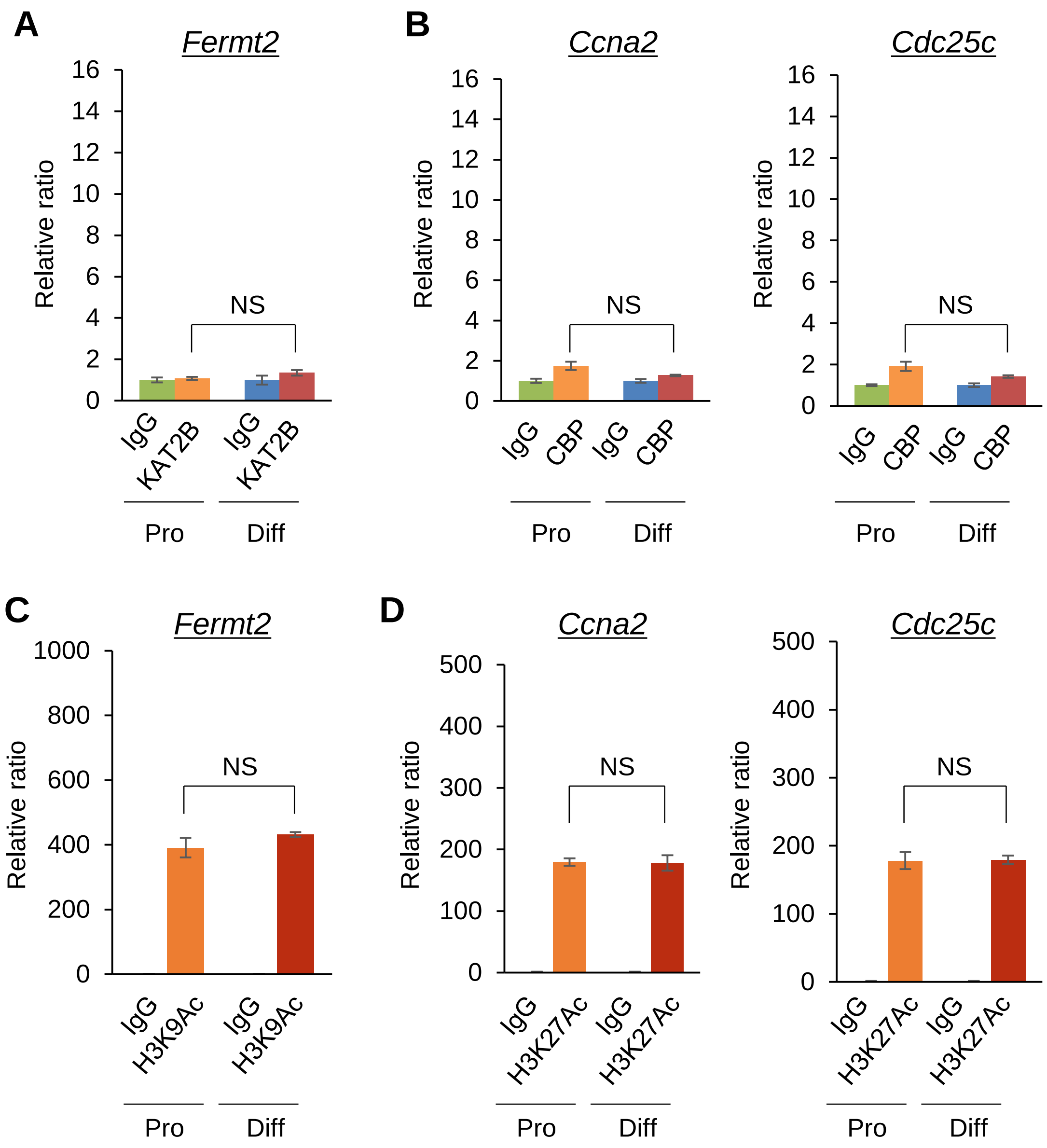
A
Fermt2
Relative ratio
16
14
12
10
8
6
4
2
0
NS
IgG
KAT2B
IgG
KAT2B
Pro
Diff
B
Ccna2
Cdc25c
IgG
CBP
IgG
CBP
C
1000
800
600
400
200
H3K9Ac
D
500
300
100
H3K27Ac

Fig. S3. Suzuki et al.

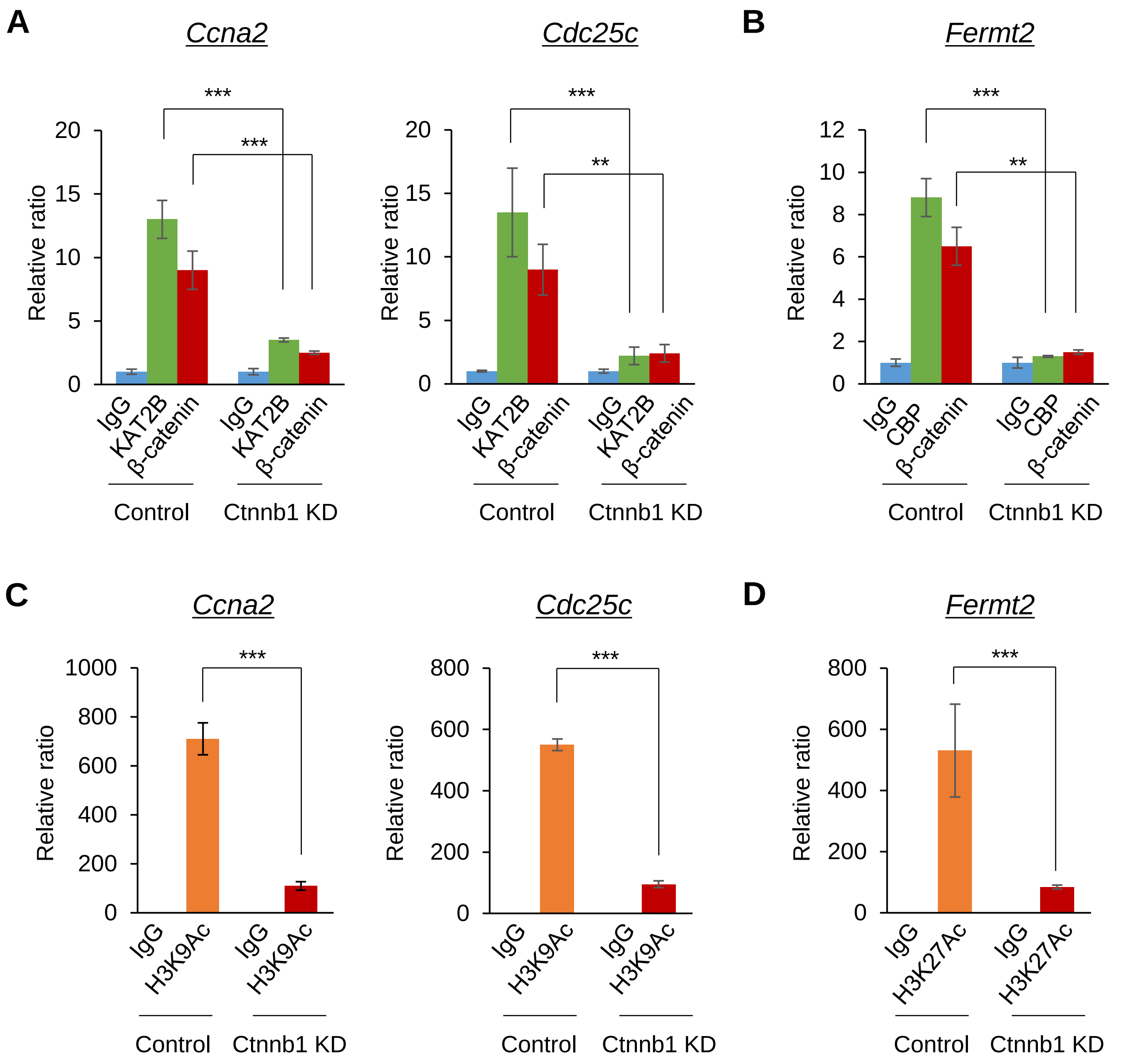

Fig. S4. Suzuki et al.

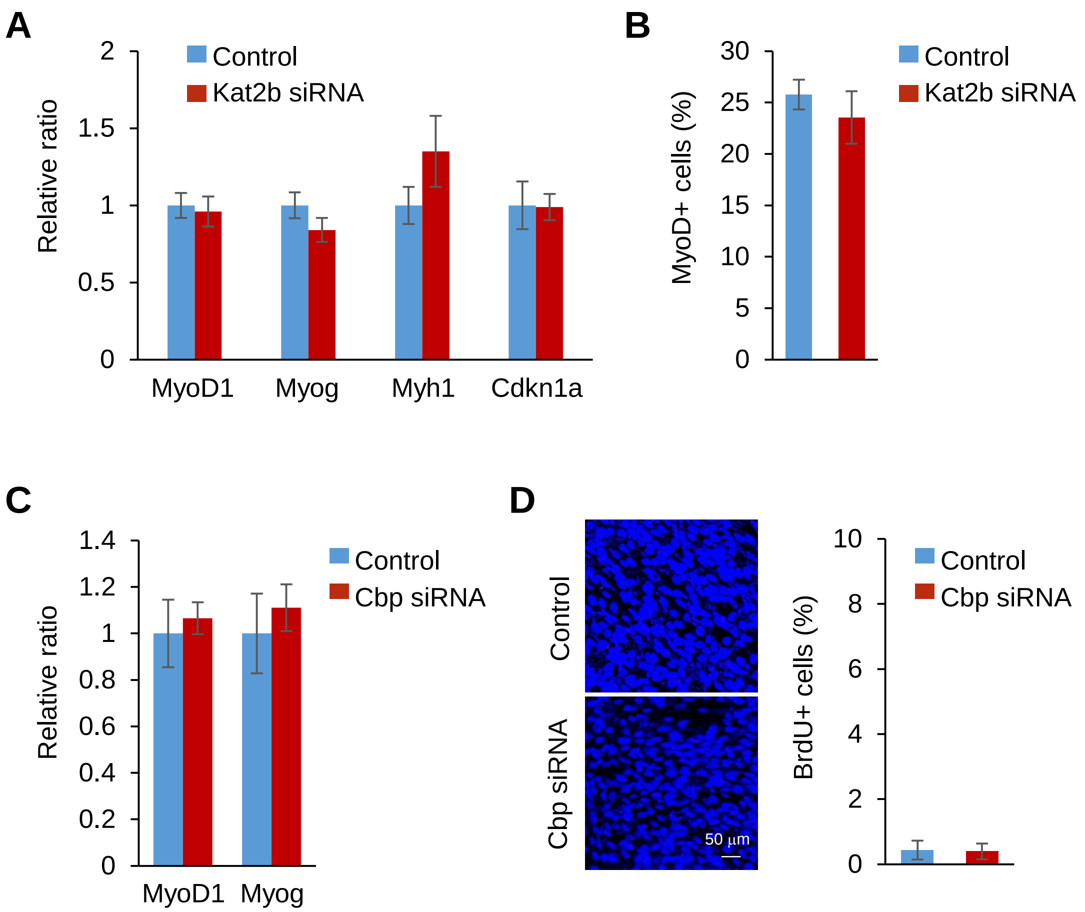

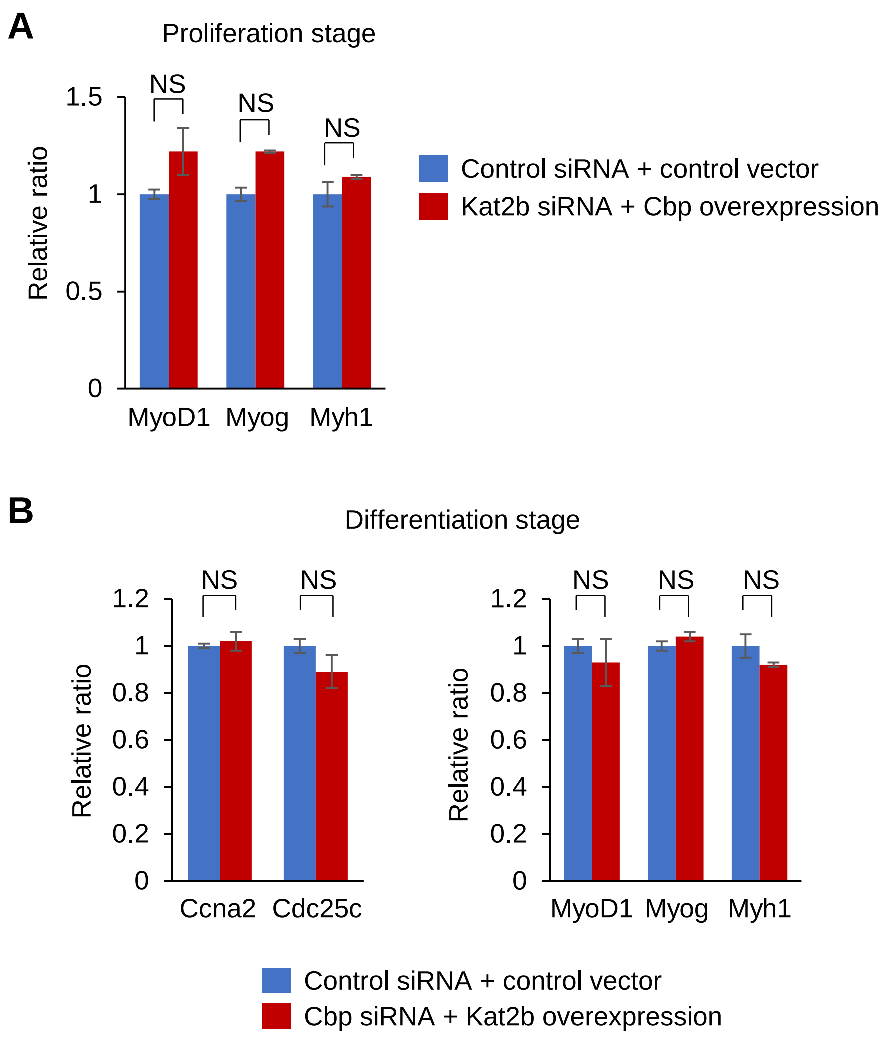
A
Proliferation stage
NS
NS
NS
1.5
1
0.5
0
Relative ratio
MyoD1 Myog Myh1
Control siRNA + control vector
Kat2b siRNA + Cbp overexpression
B
Differentiation stage
NS
NS
1.2
1
0.8
0.6
0.4
0.2
0
Relative ratio
Ccna2 Cdc25c
NS
NS
NS
MyoD1 Myog Myh1
Control siRNA + control vector
Cbp siRNA + Kat2b overexpression

Fig. S6. Suzuki et al.

Fig.2B

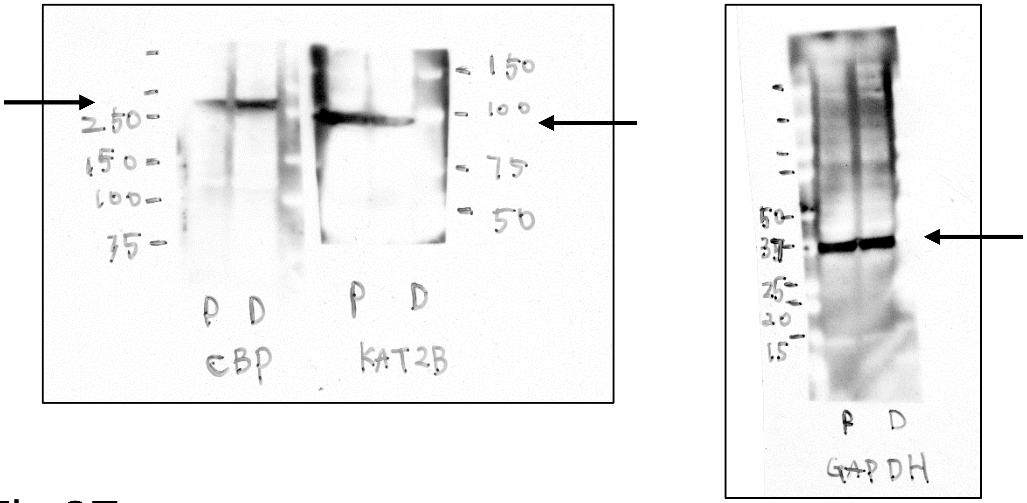

Fig.3E

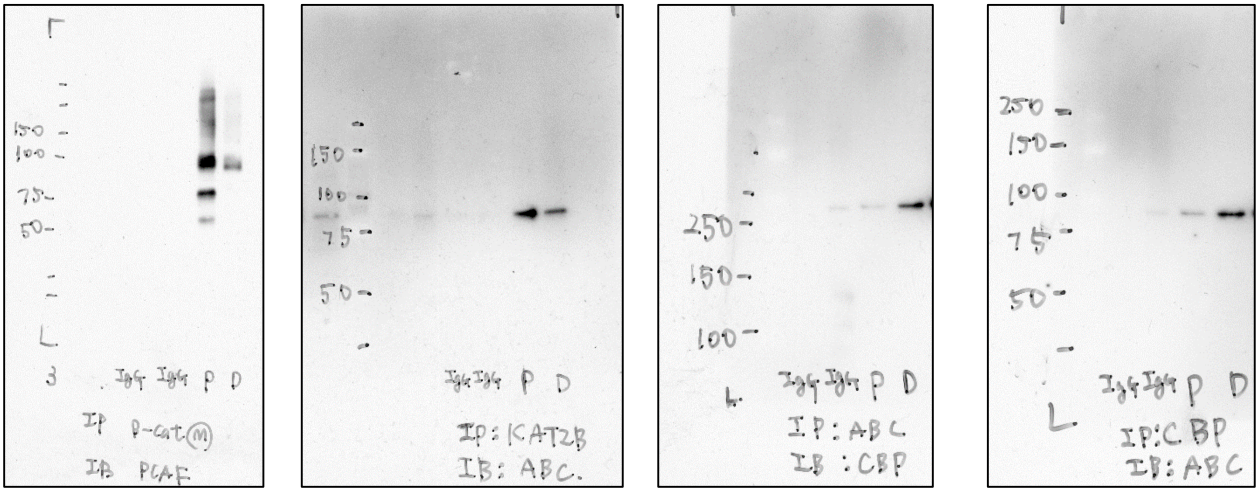

IP:β-catenin
IB: KAT2B

IP: KAT2B
IB:β-catenin

IP:β-catenin
IB: CBP

IP: CBP
IB:β-catenin

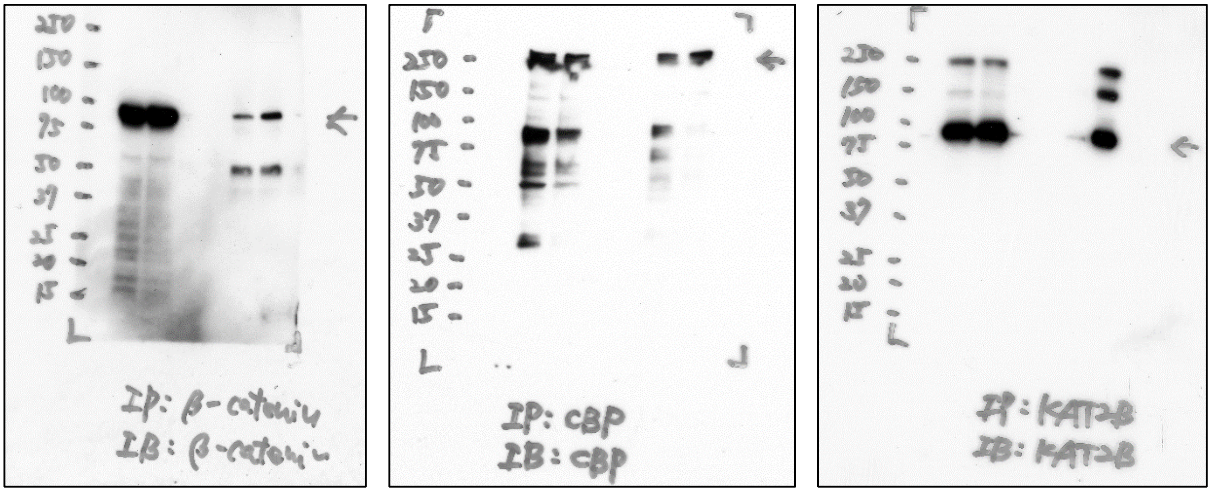

Supplement: Supplementary file 1 — Supplemental Information [file 41598_2018_32888_MOESM1_ESM.pdf]
